# The EMIF-AD Multimodal Biomarker Discovery study: design, methods and cohort characteristics

**DOI:** 10.1186/s13195-018-0396-5

**Published:** 2018-07-06

**Authors:** Isabelle Bos, Stephanie Vos, Rik Vandenberghe, Philip Scheltens, Sebastiaan Engelborghs, Giovanni Frisoni, José Luis Molinuevo, Anders Wallin, Alberto Lleó, Julius Popp, Pablo Martinez-Lage, Alison Baird, Richard Dobson, Cristina Legido-Quigley, Kristel Sleegers, Christine Van Broeckhoven, Lars Bertram, Mara ten Kate, Frederik Barkhof, Henrik Zetterberg, Simon Lovestone, Johannes Streffer, Pieter Jelle Visser

**Affiliations:** 1 Alzheimer Centrum Limburg, Maastricht University, Maastricht, the Netherlands; 2 University Hospital Leuven, Leuven, Belgium; 3 Laboratory for Cognitive Neurology, Department of Neurosciences, KU Leuven, Leuven, Belgium; 4 Alzheimer Center and Department of Neurology, VU University Medical Center, Amsterdam, the Netherlands; 5 Reference Center for Biological Markers of Dementia (BIODEM), University of Antwerp, Antwerp, Belgium; 6 Department of Neurology and Memory Clinic, Hospital Network Antwerp (ZNA) Middelheim and Hoge Beuken, Antwerp, Belgium; 7 University of Antwerp, Antwerp, Belgium; 8 University of Geneva, Geneva, Switzerland; 9 IRCCS Instituto Centro San Giovanni di Dio Fatebenefratelli, Brescia, Italy; 10 Alzheimer’s Disease & Other Cognitive Disorders Unit, Hospital Clínic—IDIBAPS, Barcelona, Spain; 11 Barcelona Beta Brain Research Center, Fundació Pasqual Maragall, Barcelona, Spain; 12 Institute of Neuroscience and Physiology, Moelndal, Sweden; 13 Hospital de la Santa Creu i Sant Pau, Barcelona, Spain; 14 Geriatric Psychiatry, Department of Mental Health and Psychiatry, Geneva University Hospitals, Geneva, Switzerland; 15 Department of Psychiatry, University Hospital of Lausanne, Lausanne, Switzerland; 16 Center for Research and Advanced Therapies, CITA—Alzheimer Foundation, San Sebastian, Spain; 17 University of Oxford, Oxford, UK; 18 King’s College London, London, UK; 19 NIHR Biomedical Research Centre for Mental Health and Biomedical Research Unit for Dementia at South London and Maudsley NHS Foundation, London, UK; 20 Farr Institute of Health Informatics Research, UCL Institute of Health Informatics, University College London, London, UK; 21 NIHR University College London Hospitals Biomedical Research Centre, London, UK; 22 Neurodegenerative Brain Diseases Group, VIB—Department of Molecular Genetics, Antwerp, Belgium; 23 Institute Born-Bunge, University of Antwerp, Antwerp, Belgium; 24 Lübeck Interdisciplinary Platform for Genome Analytics, University of Lübeck, Lübeck, Germany; 25 School of Public Health, Imperial College London, London, UK; 26 Department of Psychology, University of Oslo, Oslo, Norway; 27 Department of Radiology and Nuclear Medicine, VU University Medical Center, Amsterdam, the Netherlands; 28 Department of Psychiatry and Neurochemistry, University of Gothenburg, Mölndal, Sweden; 29 Clinical Neurochemistry Laboratory, Sahlgrenska University Hospital, Mölndal, Sweden; 30 Department of Molecular Neuroscience, UCL Institute of Neurology, London, UK; 31 UK Dementia Research Institute, London, UK; 32 Experimental Medicine, Janssen Pharmaceutical Companies, Beerse, Belgium; 33 Department of Psychiatry & Neuropsychology, School for Mental Health and Neuroscience, Alzheimer Center Limburg, Maastricht University, Universiteitssingel 40, Box 34, P.O. Box 616, 6200 MD Maastricht, the Netherlands

**Keywords:** Alzheimer’s disease, Biomarkers, Multimodal, Proteomics, Genomics, Metabolomics, Plasma, Magnetic resonance imaging, DNA, Cerebrospinal fluid

## Abstract

**Background:**

There is an urgent need for novel, noninvasive biomarkers to diagnose Alzheimer’s disease (AD) in the predementia stages and to predict the rate of decline. Therefore, we set up the European Medical Information Framework for Alzheimer’s Disease Multimodal Biomarker Discovery (EMIF-AD MBD) study. In this report we describe the design of the study, the methods used and the characteristics of the participants.

**Methods:**

Participants were selected from existing prospective multicenter and single-center European studies. Inclusion criteria were having normal cognition (NC) or a diagnosis of mild cognitive impairment (MCI) or AD-type dementia at baseline, age above 50 years, known amyloid-beta (Aβ) status, availability of cognitive test results and at least two of the following materials: plasma, DNA, magnetic resonance imaging (MRI) or cerebrospinal fluid (CSF). Targeted and untargeted metabolomic and proteomic analyses were performed in plasma, and targeted and untargeted proteomics were performed in CSF. Genome-wide SNP genotyping, next-generation sequencing and methylation profiling were conducted in DNA. Visual rating and volumetric measures were assessed on MRI. Baseline characteristics were analyzed using ANOVA or chi-square, rate of decline analyzed by linear mixed modeling.

**Results:**

We included 1221 individuals (NC *n* = 492, MCI *n* = 527, AD-type dementia *n* = 202) with a mean age of 67.9 (SD 8.3) years. The percentage Aβ+ was 26% in the NC, 58% in the MCI, and 87% in the AD-type dementia groups. Plasma samples were available for 1189 (97%) subjects, DNA samples for 929 (76%) subjects, MRI scans for 862 (71%) subjects and CSF samples for 767 (63%) subjects. For 759 (62%) individuals, clinical follow-up data were available. In each diagnostic group, the *APOE* ε4 allele was more frequent amongst Aβ+ individuals (*p* < 0.001). Only in MCI was there a difference in baseline Mini Mental State Examination (MMSE) score between the A groups (*p* < 0.001). Aβ+ had a faster rate of decline on the MMSE during follow-up in the NC (*p* < 0.001) and MCI (*p* < 0.001) groups.

**Conclusions:**

The characteristics of this large cohort of elderly subjects at various cognitive stages confirm the central roles of Aβ and *APOE* ε4 in AD pathogenesis. The results of the multimodal analyses will provide new insights into underlying mechanisms and facilitate the discovery of new diagnostic and prognostic AD biomarkers. All researchers can apply for access to the EMIF-AD MBD data by submitting a research proposal via the EMIF-AD Catalog.

**Electronic supplementary material:**

The online version of this article (10.1186/s13195-018-0396-5) contains supplementary material, which is available to authorized users.

## Background

Over the last decade great progress has been made in diagnosing Alzheimer’s disease (AD) at an early disease stage, including before the onset of dementia [1, 2]. The biomarkers amyloid-beta (Aβ) and tau in cerebrospinal fluid (CSF) or amyloid and tau load via positron emission tomography (PET) have become indispensable in the AD research field, especially as part of clinical trials for disease modification and secondary prevention [3–6]. Nonetheless, a better understanding of the underlying pathophysiological disease mechanisms as well as the discovery of diagnostic and prognostic markers that are inexpensive and minimally invasive to obtain would enhance the development of therapeutic interventions.

Currently, CSF and PET biomarkers are commonly used for the early diagnosis and prognosis of AD [7–9]. However PET imaging is fairly expensive and not universally available and the procedure for obtaining a PET scan as well as CSF data are relatively invasive. Given this, complementing these highly specific biomarker modalities with markers in more readily accessible biofluids would mark an important step forward. Consequently, many initiatives have been undertaken to discover and validate blood-based biomarkers for AD pathology [10, 11], but so far results have been limited, due to small sample sizes, single modality analyses or other methodological issues [12]. One critical issue so far has been the design (comparing individuals with AD-type dementia with controls), which made the studies unsuitable for discovery of markers for the preclinical disease phase. To seek markers for the preclinical phase, a more sensitive and gradual approach has been proposed, described as the “endophenotype approach” where discovery is predicted on a measure of pathology [13]. Therefore, we designed the current study to enhance blood-based biomarker discovery by performing a series of omics techniques (e.g., proteomics, metabolomics, genomics) in a large cohort across the AD clinical disease spectrum, using an endophenotype approach.

This study was performed as a part of the European Medical Information Framework for Alzheimer’s disease (EMIF-AD; http://www.emif.eu). Funded through the Innovative Medicines Initiative (IMI), the EMIF project was established to facilitate the process of reusing and combining existing healthcare data with a focus on two therapeutic areas in the first instance: metabolic diseases and AD. One of the main aims of the EMIF-AD project is to accelerate the discovery of novel diagnostic and prognostic biomarkers for AD and to unravel the underlying pathophysiological mechanisms, using existing data and existing samples, that would otherwise be inaccessible to research beyond the project teams responsible for the collection. In this report, we will describe the set-up of the EMIF-AD Multimodal Biomarker Discovery (EMIF-AD MBD) study, the methods as well as the characteristics of the included subjects. The results of the single and multimodal analyses will be described in future publications.

## Methods

### General outline

In the EMIF-AD MBD study we retrospectively combined and reused clinical data, samples and scans that had already been collected as part of existing prospective cohort studies. We aimed to include a total of 1000 subjects across the clinical AD spectrum: 400 subjects with normal cognition (NC), 400 subjects with mild cognitive impairment (MCI) and 200 subjects with mild AD-type dementia. To create a balanced design in terms of progression and to enable endophenotype designed biomarker studies, we intended to include 50% Aβ-positive (Aβ+) individuals and 50% Aβ-negative (Aβ–) individuals in the groups with NC and MCI. To conduct multimodal analyses, we initially aimed to include subjects who had material from MRI, plasma, DNA and CSF. Later, we adjusted this to subjects with material available in at least two of the modalities listed.

### Selection of cohorts

**Fig. 1:**
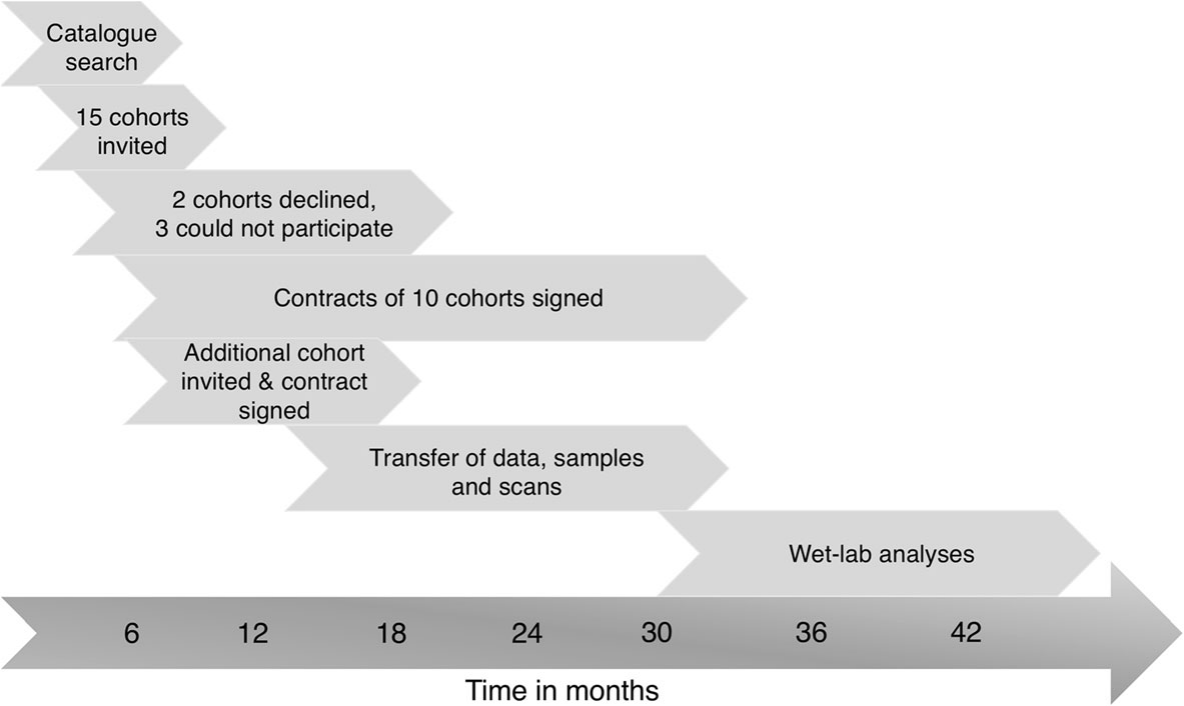
Timeline of events related to the EMIF-AD MBD study

We used the EMIF-AD Catalog (https://emif-catalogue.eu), established as part of the objective of the EMIF which seeks to enable the finding, assessment and reutilization of preexisting data. The EMIF-AD Catalog contains metadata about European AD cohorts, enabling the selection of studies that included subjects who, in this instance, met the following inclusion criteria: data on Aβ status, measured in CSF or by amyloid positron emission tomography (PET); age above 50 years at baseline; and availability of MRI scans, plasma and DNA samples. We identified 16 suitable cohorts. Two cohorts declined due to other research interests. Three cohorts were interested to collaborate, but unable because of legal and/or ethical restrictions, or unavailability of sufficient sample volumes. The 11 selected cohorts included three multicenter studies—EDAR (*n* = 204) [14], PharmaCog (*n* = 147) [15] and DESCRIPA (*n* = 29) [16]—and eight single centers: Antwerp (*n* = 149) [17], Amsterdam (*n* = 172) [18], Barcelona Sant Pau (*n* = 45) [19], Barcelona IDIBAPS (*n* = 120) [20], Leuven (*n* = 180) [21], San Sebastian GAP (*n* = 40) [22], Gothenburg (*n* = 95) [23] and Lausanne (*n* = 40) [24]. Of these 11 cohorts, DESCRIPA, EDAR, PharmaCog, Amsterdam, Antwerp and Gothenburg were linked to partners in the EMIF-AD, while the other five cohorts participated as affiliated data providers (ADP). All cohorts (e.g., partners and ADP) signed a material transfer agreement. The ADP also agreed to the EMIF project agreement. Study managers from each cohort selected the subjects based on the following criteria: age above 50 years at baseline; availability of Aβ status at baseline measured in CSF or via PET; availability of neuropsychological and clinical data; availability of at least two of the following materials: MRI scan, plasma sample, DNA samples or CSF sample; and absence of neurological, psychiatric or somatic disorders that could cause cognitive impairment. The local medical ethical committee in each center approved the study. Subjects had already provided written informed consent at the time of inclusion in the cohort for use of data, samples and scans. Figure 1 shows a timeline of the different events in establishing this cohort, from the search in the EMIF Catalog to the wet-lab analyses.

### Baseline diagnoses

In all cohorts, the definition for NC was a normal performance on neuropsychological assessment (within 1.5 SD of the average for age, gender and education). Five cohorts also used a score of 0 on the Clinical Dementia Rating (CDR) [25] and a single cohort used a cutoff value < 3 on the Global Deterioration Scale [26] to determine NC. Diagnosis of MCI was made according to the criteria of Petersen [27] in nine cohorts. Two cohorts used the Winblad et al. criteria [28] to diagnose MCI. All cohorts used the National Institute of Neurological and Communicative Disorders and Stroke–Alzheimer’s Disease and Related Disorders Association criteria (NINCDS-ADRDA) criteria [29] to diagnose AD-type dementia. Additional file 1: Table S1 presents the diagnostic criteria used per center.

### Clinical data

All cohorts were asked to contribute available data on demographics, clinical information, neuropsychological testing and Aβ status, as presented in Table 1. Medication use and comorbidities were classified into a number of categories, for which we created dichotomous variables (Table 1).

**Table 1:** Clinical dataset

**Demographics**	
Age	
Gender	
Years of education	
**Clinical information**	
Diagnosis	
Medication use	
Cardiovascular medication	
Dementia medication	
Hormonal medication	
Psychopharmaceuticals	
Other medication	
Comorbidities	
Cardiovascular disorders	
Cardiovascular risk factors	
Cerebrovascular disorders	
Endocrine disorders	
Neurological disorders	
Other cardiac disorders	
Psychiatric disorders	
Somatic disorders	
Family history of dementia	
First-degree relatives	
Second-degree relatives	
Functional impairment rating	
**Neuropsychological tests**	
Memory, preferred test: AVLT	
Language, preferred test: animal fluency	
Attention, preferred test: Trail Making Test A	
Executive functioning, preferred test: Trail Making Test B	
Visuoconstruction, preferred test: Rey complex figure copy	
**Aβ measure** ^**a**^	
CSF Aβ_42_ value and local cutoff point	
Amyloid PET SUV and local cutoff point	

^a^At least one Aβ measure

*Aβ* amyloid-beta, *AVLT* Auditory Verbal Learning Test, *CSF* cerebrospinal fluid, *PET* positron emission tomography, *SUV* standardized uptake value

Cognitive data were collected in all cohorts. The cognitive tests used varied across centers. Only the Mini Mental State Examination (MMSE) was administered in all centers and was available for nearly all subjects (*n* = 1216). We requested at least one test from the following cognitive domains: memory, language, attention, executive functioning and visuoconstruction [16]. For each cognitive domain, we selected a primary test (Table 1). If the preferred tests were not available, we selected an alternative priority test from the same cognitive domain. Additional file 2: Table S2 provides an overview of the different tests used for each cognitive domain. For each test, we requested the raw scores and, if available, *z*-scores calculated based on local normative data. If local normative data were unavailable, we calculated *z*-scores based on published normative data from healthy controls for that test. Per cognitive domain, we combined *z*-scores which we used as a continuous variable, and we used a cutoff value of *z*-score < − 1.5 to define abnormality.

Clinical data were harmonized, pooled and stored on an online data platform using tranSMART [30], now enriched for dementia research purposes through the EMIF-AD project.

### Plasma analyses

Initially, the minimum requested amount of plasma was 0.7 ml. If available, another 0.7 ml was requested to conduct additional analyses in a subgroup of subjects. In some cases, only 0.5 ml was available. Prior to the analyses, samples were checked visually for consistency and volume. Results of analyses were also quality checked by inspecting patterns of outliers, and excluding consistent outliers across analyses. Analyses conducted on these samples include: targeted analyses of plasma proteins identified previously [31] and confirmed in at least one replication study, a panel of complement proteins nominated because of increasing evidence from genomics of the role of innate immunity in AD and analysis of plasma neurofilament light (NFL) chain; untargeted proteomic analyses using aptamer capture approaches; and untargeted and targeted metabolic analyses using a 883-metabolite panel for the targeted assay.

### Genetic analyses

A total amount of 2.6 μg DNA or 1 ml whole blood, from which DNA was to be extracted, was requested for the genetic analyses. After performing routine quality checks on extracted DNA (e.g., agarose gel electrophoresis, determination of A260/280 and A260/230 ratios, PicoGreen quantification), we performed three types of assessments on each sample passing quality control: genome-wide SNP genotyping (Global Screening Array; Illumina, Inc.), genome-wide DNA methylation profiling (Infinium MethylationEPIC BeadChip; Illumina, Inc.) and whole exome sequencing.

### CSF analyses

The requested amount of CSF to conduct all planned analyses was 0.4 ml, which was used for untargeted proteomic and peptidomic analyses, and a number of targeted analyses measuring concentrations of Aβ_38_, Aβ_40_, Aβ_42_, Aβ_42/40_, YKL-40, NFL and neurogranin (Ng). Prior to the analyses, CSF samples were checked visually for volume and absence of blood contamination.

### MRI analyses

MRI scans were assembled centrally, quality checked and assessed visually by a single rater. T1-weighted and, when available, FLAIR and/or T2*/SWI images were used for qualitative visual rating, including medial temporal lobe atrophy [32], global cortical atrophy [33], white matter hyperintensities [34] and microbleeds (defined as small (< 10 mm) round foci of hypointense signal in brain parenchyma). 3D T1 scans were uploaded to the Neurgrid platform (https://neugrid4you.eu) [35] for storage and automated quantitative analyses. Volumetric analysis included assessment of hippocampal and whole brain volume and cortical thickness.

### Amyloid classification

Aβ status was defined by the CSF Aβ_42/40_ of the central analyses, using a cutoff value of < 0.061 to determine abnormality (*n* = 770). The cutoff value for the Aβ_42/40_ ratio was determined based on mixture model analyses comparing the NC and AD groups in this dataset. When no CSF was contributed for central analyses, the local CSF Aβ_42_ value (*n* = 271) or the standardized uptake value ratio (SUVR) on an amyloid PET scan (*n* = 180) with local cutoff values to determine abnormality were used (Additional file 3: Table S3).

### Statistical analyses

Baseline characteristics were compared between groups using ANOVA for continuous variables and chi-square for categorical variables. General linear mixed models with random intercepts and slopes by study were used to examine the influence of Aβ status on MMSE performance and decline over time, adjusted for age, gender and years of education. Missing values for APOE genotype (*n* = 12) and years of education (*n* = 105) were imputed using regression within study with at least two significant predictors (i.e., age, gender, MMSE, etc.). Statistical analyses were performed using R Statistical Software (version 3.3.3) and SPSS (version 24), with significance defined as *p* < 0.05.

## Results

We initially sought to identify 1000 individuals with data available in all modalities. However, because not all studies could contribute data for all modalities, we included more participants to meet the aimed number of individuals for each modality. In total, 1221 subjects were included in the study, with a mean age of 67.9 (SD 8.3) years. Six hundred and sixty-seven (54%) were female and the average education level was 11.7 (SD 4.1) years. At baseline, 492 (40%) subjects had NC, 527 (43%) subjects had a diagnosis of MCI and 202 (17%) subjects had a clinical diagnosis of AD-type dementia. For 758 (62%) individuals there were follow-up data available (e.g., at least a clinical diagnosis or MMSE at follow-up): 217 (44%) NC subjects, 398 (76%) MCI subjects and 143 (71%) demented subjects. The average follow-up time for all 758 individuals was 2.3 (SD 1.2) years. Per diagnostic groups, the average clinical follow-up time was: NC 2.4 (SD 0.9) years, MCI 2.2 (SD 1.3) years and AD 2.2 (SD 1.4) years.

Table 2 presents the baseline characteristics of the sample by Aβ status and by baseline diagnosis. In the NC and MCI groups, the Aβ+ subjects were older than the Aβ– subjects (NC, *p* = 0.002; MCI, *p* < 0.001). In all diagnostic groups, Aβ+ subjects were more likely to be an APOE ε4 carrier (all *p* < 0.001). In the MCI subjects only, there was a difference in baseline MMSE score between the Aβ groups (*p* = 0.001). Regarding cognitive domains, we found differences in memory (*p* < 0.001) and executive functioning (*p* = 0.042) *z*-scores in individuals with MCI. In individuals with AD-type dementia we found that Aβ+ individuals performed worse on an executive functioning task (*p* = 0.013).

**Table 2:** Baseline characteristics by clinical diagnosis and Aβ status

		Normal cognition		MCI		AD-type dementia	
		Aβ–	Aβ+	Aβ–	Aβ+	Aβ–	Aβ+
	Total *n*	*n* = 365	*n* = 127	*n* = 220	*n* = 307	*n* = 27	*n* = 175
Age (years)	1221	64.4 (7.6)	66.9 (7.9)**	68.3 (8.2)	70.7 (7.4)***	73.0 (8.4)	69.9 (8.8)
Female, *n*	1221	203 (56)	66 (52)	108 (49)	172 (56)	12 (44)	96 (55)
Education (years)	1221	13.5 (3.7)	12.7 (4.0)*	10.6 (3.8)	10.8 (3.7)	8.5 (4.4)	10.6 (3.8)**
APOE ε4 carrier, *n*	1221	122 (33)	76 (60)***	46 (21)	200 (65)***	7 (26)	114 (65)***
Mean follow-up time (years)	758	2.3 (0.8)	2.5 (1.1)	2.2 (1.3)	2.2 (1.3)	1.7 (0.9)	2.2 (1.4)
MMSE score	1215	28.9 (1.1)	28.8 (1.2)	27.0 (2.3)	25.9 (2.7)***	21.5 (5.4)	21.7 (4.6)
Memory delayed *z*-score	1049	0.1 (1.1)	0.0 (1.2)	−0.9 (1.3)	−1.4 (1.4)***	−2.2 (1.2)	−2.4 (1.1)
Language *z*-score	1181	−0.2 (1.0)	−0.1 (1.0)	− 0.7 (1.2)	− 1.0 (2.0)	− 1.9 (1.2)	− 2.3 (2.4)
Attention *z*-score	1128	0.3 (1.1)	0.2 (0.9)	−1.0 (1.8)	−1.0 (1.8)	−2.1 (2.5)	−2.1 (2.0)
Executive functioning *z*-score	976	0.3 (1.1)	0.1 (1.1)	−0.9 (1.9)	−1.4 (2.1)*	−1.2 (2.5)	−3.4 (2.8)*
Visuoconstruction *z*-score	664	0.2 (1.4)	0.2 (0.8)	−0.3 (1.7)	−0.4 (1.8)	−2.1 (2.4)	−1.3 (2.0)

Results are mean (standard deviation) for continuous variables or frequency (%) for dichotomous variables

*Aβ* amyloid-beta, *AD* Alzheimer’s disease, *APOE* apolipoprotein E, *MCI* mild cognitive impairment, *MMSE* Mini Mental State Examination

*p < 0.05 in comparison to Aβ– group

**p < 0.01in comparison to Aβ– group

***p < 0.001 in comparison to Aβ– group

Table 3 presents the number of subjects per modality by diagnostic category. Plasma samples were contributed for 1189 (97%) subjects, DNA for 929 (76%) subjects, MRI scans for 862 (71%) subjects and CSF for 770 (63%) subjects. There were 482 (40%) subjects who contributed material in all modalities. Of this subsample, 89 (18%) subjects had NC, 318 (66%) subjects MCI and 75 (16%) subjects had a diagnosis of AD-type dementia at baseline.

**Table 3:** Number of subjects from different cohorts for each modality by diagnosis

Cohort	Diagnosis	Clinical data	Plasma	DNA	MRI	CSF
Amsterdam	NC	30	29	26	30	30
	MCI	82	80	68	82	82
	AD-type dementia	60	60	53	60	60
Antwerp	MCI	103	100	101	50	103
	AD-type dementia	46	47	46	0	46
DESCRIPA	NC	12	12	8	5	12
	MCI	17	17	12	9	17
EDAR	NC	48	47	42	14	47
	MCI	77	75	65	24	75
	AD-type dementia	79	78	69	19	76
GAP	NC	40	40	40	38	40
Gothenburg	NC	49	48	–	48	–
	MCI	46	44	–	46	–
IDIBAPS	NC	76	77	–	40	–
	MCI	27	27	–	14	–
	AD-type dementia	17	16	–	14	–
Lausanne	NC	12	12	12	12	12
	MCI	28	28	28	27	28
Leuven	NC	180	163	168	179	–
PharmaCog	MCI	147	144	146	147	147
Sant Pau	NC	45	45	45	–	–
Total	NC	492	473	341	366	141
	MCI	527	515	420	399	452
	AD-type dementia	202	201	168	100	182
	Overall	1221	1189	929	865	775

*CSF* cerebrospinal fluid, *DESCRIPA* development of screening guidelines and clinical criteria for predementia Alzheimer's disease, *EDAR* beta amyloid oligomers in the early diagnosis of AD and as a marker for treatment reponse, *GAP* gipuzkoa Alzheimer project, *IDIBAPS* institut d'investigacions biomèdiques August Pi i Sunyer, *MCI* mild cognitive impairment, *MRI* magnetic resonance imaging, *NC* normal cognition

**Fig. 2:**
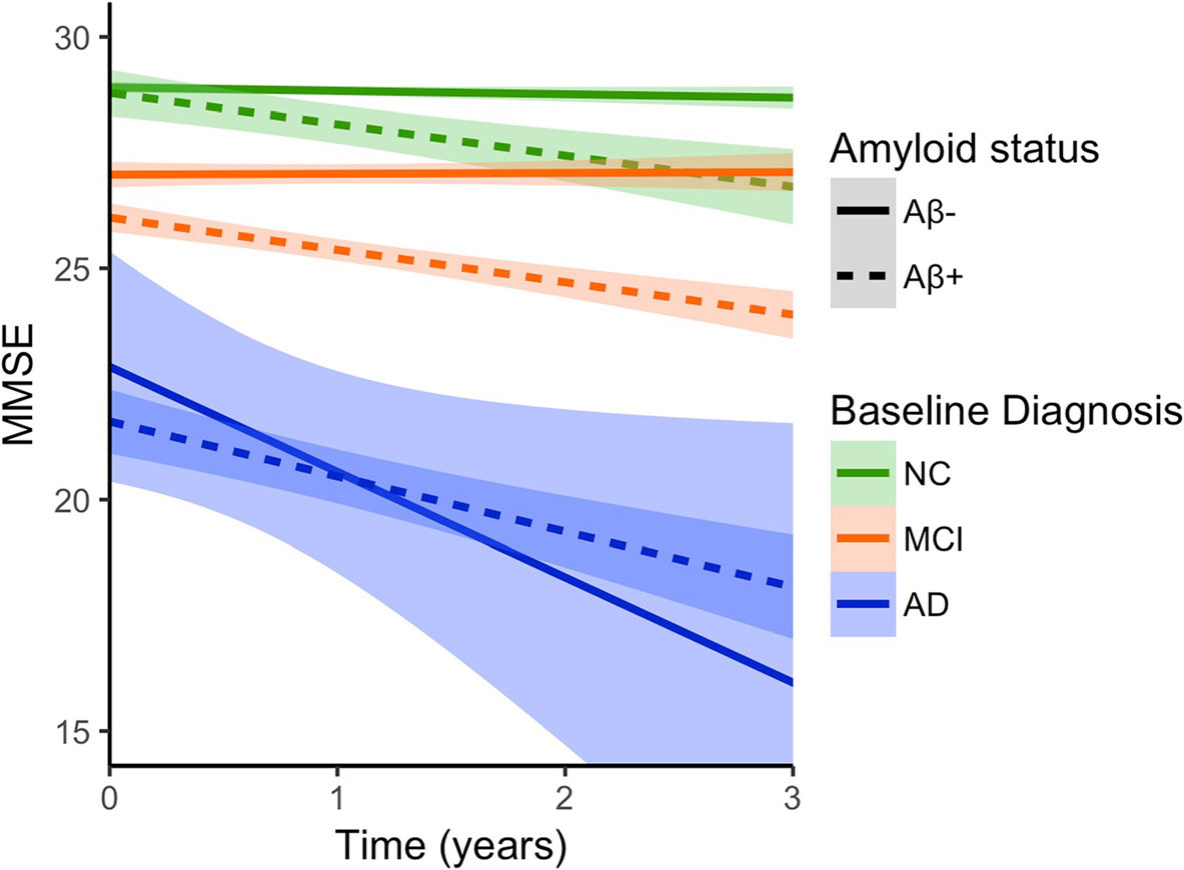
MMSE scores over time by Aβ status and diagnosis. Mean scores and 95% confidence intervals of MMSE over time for three diagnostic groups and by Aβ status, adjusted for demographics. Solid lines represent Aβ– groups, dashed lines represent Aβ+ groups. Aβ amyloid-beta, AD Alzheimer’s disease, MCI mild cognitive impairment, MMSE Mini Mental State Examination, NC normal cognition

Table 4 and Fig. 2 show the effect of Aβ on MMSE scores over time for each diagnostic group, adjusted for demographics. At baseline, there is only a difference in MMSE for the MCI group (*p* < 0.001). In the NC and MCI groups, the Aβ+ individuals in the NC and MCI groups decline at a faster rate than the Aβ– individuals (NC, *p* < 0.001; MCI, *p* < 0.001). For the demented subjects, Aβ did not influence the rate of decline (Table 4, Fig. 2).

**Table 4:** Effect of Aβ on MMSE scores over time by diagnostic group

Diagnosis	*n*	Baseline	*p* value	Slope	*p* value
NC	482	− 0.35 ± 0.20	0.170	− 0.60 ± 0.13	< 0.001
MCI	459	− 1.56 ± 0.24	< 0.001	− 0.60 ± 0.14	< 0.001
AD dementia	162	− 0.05 ± 1.05	0.965	− 0.21 ± 0.64	0.742

Numbers are linear mixed-model coefficients ± standard error, relative to the Aβ– group, adjusted for age, gender and years of education

*Aβ* amyloid-beta, *AD* Alzheimer’s disease, *MCI* mild cognitive impairment, *MMSE* Mini Mental State Examination, *NC* normal cognition

## Discussion

The aim of the EMIF consortium is to enable the reutilization of preexisting data including the finding and assessment of relevant datasets and facilitation of their interoperability and reuse. For the EMIF-AD component, a major use-case objective has been to use the processes and tools established in the consortium to generate a novel cross-cohort data and sample collection for the discovery and validation of biomarkers for use in clinical trials using a multimodal and endophenotype design. The first results presented in this report confirm the central roles of Aβ and *APOE* ε4 in the pathogenesis of AD, which is consistent with findings from other large cohorts [36, 37]. The molecular studies are ongoing and will be reported in future publications.

AD is a complex and multifactorial disorder, which underscores the need for multimodal studies with sufficient statistical power [38]. To date these large studies are scarce, especially those including subjects across the whole clinical AD spectrum. To our knowledge, the only other large-scale studies that collected plasma, DNA, CSF and imaging material from individuals in various cognitive stages are the Alzheimer’s Disease Neuroimaging Initiative (ADNI) [36] and the Australian Imaging, Biomarkers and Lifestyle (AIBL) study of aging [37] studies. Since these datasets are so unique, findings from these studies are difficult to validate. The current study will not only be of great additional value due to its explorative nature and complementary laboaratory analyses, but also because previous findings can be validated in a large-size cohort with multimodal data. We collected a wide variety of clinical variables including neuropsychological tests, comorbidities, medication use and psychiatric questionnaires. All of the clinical data and results from the multimodal wet-lab analyses will be stored on an online, secure data platform (tranSMART). Research proposals can be submitted via the EMIF-AD Catalog (https://emif-catalogue.eu) to work with these data, which require approval from the EMIF-AD team and the data-owners.

Besides the major advantages, this study also has some limitations. Currently, we do not have clinical follow-up data for all subjects, as some centers are still in the process of collecting these. However, these data may be added to the database in the future. Also, the data, samples and scans contributed to this study were collected at different centers and were not collected using the same protocol, which will lead to preanalytical variability. To limit this variability, the samples were analyzed centrally and the clinical data were harmonized using standardized values and dichotomous variables.

## Conclusion

The various complementary analyses conducted in plasma, DNA and CSF and on MRI scans in a large-sized cohort of individuals across the clinical AD spectrum provide a unique opportunity to discover novel diagnostic and prognostic markers, and will also increase knowledge into the AD pathophysiology, which is required for the development of novel therapeutic interventions.

## Additional files

Additional file 1: **Table S1.** Diagnostic criteria per cohort. Cohorts, countries, number of subjects and diagnostic criteria used for NC, MCI and AD dementia (DOCX 90 kb)

Additional file 2: **Table S2.** Number of subjects per test by cognitive domain at baseline. Number of subjects per test and norms used in domains of global cognition, memory, language, attention, executive functioning and visuoconstruction (DOCX 90 kb)

Additional file 3: **Table S3.** Biomarker protocol information for each cohort. Protocol information for PET, CSF and plasma biomarker collection (DOCX 100 kb)

Additional file 4: **Table S4.** Ethical approval committee of each center. Ethical approval committees in each of the participating centers (DOCX 109 kb)

## Abbreviations

AD: Alzheimer’s disease
ADP: Affiliated data provider
APOE: Apolipoprotein E
Aβ: Amyloid-beta
CSF: Cerebrospinal fluid
IMI: Innovative Medicine Initiative
MCI: Mild cognitive impairment
MMSE: Mini Mental State Examination
MRI: Magnetic resonance imaging
NC: Normal cognition
NFL: Neurofilament light
Ng: Neurogranin
PET: Positron emission tomography
