# Supplementary material for: The EMIF-AD Multimodal Biomarker Discovery study: design, methods and cohort characteristics
Source: Alzheimers Res Ther. 2018 Jul 6;10:64. doi: 10.1186/s13195-018-0396-5 (PMC6035398; doi:10.1186/s13195-018-0396-5)
Supplement: Supplementary file 1 — Table S1. Diagnostic criteria per cohort. Cohorts, countries, number of subjects and diagnostic criteria used for NC, MCI and AD dementia (DOCX 90 kb) [file 13195_2018_396_MOESM1_ESM.docx]

| **Diagnostic criteria per cohort** | | | | | |
| --- | --- | --- | --- | --- | --- |
|  |  |  | Diagnostic criteria | | |
| Cohort | Country | n | CN | MCI | AD-type dementia |
| Amsterdam | The Netherlands | 172 | >-1.5 SD on NPE and CDR=0 or 0.5 | Petersen criteria^1^ | NINCDS-ADRA^3^ |
| Antwerp | Belgium | 149 | >-1.5 SD on NPE | Petersen criteria^1^ | NINCDS-ADRA^3^ |
| DESCRIPA | European multicenter | 29 | >-1.5 SD on NPE | Petersen criteria^1^ | NINCDS-ADRA^3^ |
| EDAR | European multicenter | 204 | >-1.5 SD on NPE | Petersen criteria^1^ | NINCDS-ADRA^3^ |
| GAP | Spain | 40 | >-1.5 SD on NPE and CDR=0 | Petersen criteria^1^ | NINCDS-ADRA^3^ |
| Gothenburg | Sweden | 95 | >-1.5 SD on NPE | Winblad criteria^2^ | NINCDS-ADRA^3^ |
| IDIBAPS | Spain | 120 | >-1.5 SD on NPE and CDR=0 | Petersen criteria^1^ | NINCDS-ADRA^3^ |
| Lausanne | Switzerland | 40 | >-1.5 SD on NPE and CDR=0 | Winblad criteria^2^, CDR=0.5 | NINCDS-ADRA^3^ |
| Leuven | Belgium | 180 | >-1.5 SD on NPE, CDR=0 and MMSE>27 | Petersen criteria^1^ | NINCDS-ADRA^3^ |
| Pharmacog | European multicenter | 147 | >-1.5 SD on NPE | Petersen criteria^1^, CDR=0.5 | NINCDS-ADRA^3^ |
| Sant Pau | Spain | 45 | >-1.5 SD on NPE and GDS<3 | Petersen criteria^1^, GDS=3 | NINCDS-ADRA^3^ |
| AD = Alzheimer’s disease, CDR = Clinical Dementia Rating, CN = Cognitively normal, DSM-IV = Diagnostic and Statistical Manual of Mental Disorders, GDS = global deterioration scale, MCI = Mild Cognitive Impairment, MMSE = Mini Mental State Examinations, NINCDS-ADRA = National Institute of Neurological and Communicative Disorders and Stroke – Alzheimer’s Disease and Related Disorders Association; NPE = Neuropsychological Examination; ^1^Petersen RC: Mild Cognitive Impairment as a diagnostic entity. *Journal of internal medicine* 2004; ^2^Winblad B, Palmer K, Kivipelto, M. et al. Mild cognitive impairment – beyond controversies, towards a consensus: report of the International Working Group on Mild Cognitive Impairment. *Journal of internal medicine.* 2004; ^3^ McKhann G., Drachman, D., Folstein, M., et al. Clinical diagnosis of Alzheimer’s disease: report of the NINCDS-ADRDA Work Group under auspices of Department of Health and Human Services Task Force on Alzheimer’s Disease. *Neurology*, 1984 | | | | | |
