# Supplementary material for: The EMIF-AD Multimodal Biomarker Discovery study: design, methods and cohort characteristics
Source: Alzheimers Res Ther. 2018 Jul 6;10:64. doi: 10.1186/s13195-018-0396-5 (PMC6035398; doi:10.1186/s13195-018-0396-5)
Supplement: Supplementary file 2 — Table S2. Number of subjects per test by cognitive domain at baseline. Number of subjects per test and norms used in domains of global cognition, memory, language, attention, executive functioning and visuoconstruction (DOCX 90 kb) [file 13195_2018_396_MOESM2_ESM.docx]

| **Number of subjects per test by cognitive domain at baseline** | | | |
| --- | --- | --- | --- |
| Cognitive Domain | Tests | n | Normative data used |
| Global cognition | Mini Mental State Examination | 1216 |  |
| Memory | At least one test | 1197 |  |
|  | AVLT | 613 | [1, 2] and local norms |
|  | Free and Cued Selective Reminding Test | 175 | [3-5] |
|  | Word list of CERAD neuropsychological battery | 217 | [6, 7] and local norms |
|  | Wechsler Memory Scale, subscales or whole scale | 63 | [8] |
|  | RBANS, memory index | 49 | [9] |
|  | MMSE, memory items | 29 | Cut-off (<3) to define abnormality |
|  | HDS, recent memory items | 29 | Cut-off (<5) to define abnormality |
|  | RI-48 Test | 22 | Local norms |
| Language | At least one test | 1182 |  |
|  | 1-minute verbal fluency animals | 796 | [10] |
|  | Category fluency sum of three categories | 147 | [11] |
|  | Boston Naming Test | 113 | [12, 13] |
|  | 1-minute verbal fluency words starting with ‘F’, ‘A’, ‘S’ | 90 | Local norms |
|  | 2-minute verbal fluency animals | 45 | Local norms |
|  | RBANS, language index | 31 | [9] |
| Attention | At least one test | 1156 |  |
|  | Trail Making Test, part A | 1035 | [7, 14] and local norms |
|  | Stroop, part 1 | 183 | [15] and local norms |
|  | RBANS, concentration index | 47 | [9] |
|  | Wechsler Memory Scale, working memory/concentration | 40 | [8] |
|  | HDS, concentration index | 30 | Cut-off (<5) to define abnormality |
| Executive functioning | At least one test | 970 |  |
|  | Trail Making Test, part B | 957 | local |
|  | Stroop, part 3 | 15 | [15] and local norms |
| Visuoconstruction | At least one test | 719 |  |
|  | Copy of Rey complex figure | 431 | [4, 16] and local norms |
|  | Copy CERAD figures | 207 | [7] |
|  | RBANS, visuoconstruction | 49 | [9] |
|  | HDS, drawing item | 32 | Cut-off (<5) to define abnormality |
| RAVLT = Rey Auditory Verbal Learning Test, CERAD = Consortium to Establish a Registry for AD, HDS = Hasegawa Dementia Scale, MMSE = Mini Mental State Examination, RBANS = Repeatable Battery for the Assessment of Neuropsychological Status | | | |

**References for normative data**

1. Van der Elst W, van Boxtel MP, van Breukelen GJ, Jolles J: **Rey's verbal learning test: normative data for 1855 healthy participants aged 24-81 years and the influence of age, sex, education, and mode of presentation**. *Journal of the International Neuropsychological Society : JINS* 2005, **11**(3):290-302.

2. Ivnik RJ, Malec JF, Tangalos EG, Petersen RC, Kokmen E, Kurland LT: **The Auditory-Verbal Learning Test (AVLT): norms for ages 55 years and older**. *Psychological Assessment: A Journal of Consulting and Clinical Psychology* 1990, **2**(3):304.

3. Ivnik RJ, Smith GE, Lucas JA, Tangalos EG, Kokmen E, Petersen RC: **Free and cued selective reminding test: MOANS norms**. *Journal of Clinical and Experimental Neuropsychology* 1997, **19**(5):676-691.

4. Peña-Casanova J, Gramunt-Fombuena N, Quiñones-Úbeda S, Sánchez-Benavides G, Aguilar M, Badenes D, Molinuevo JL, Robles A, Barquero MS, Payno M: **Spanish multicenter normative studies (NEURONORMA Project): norms for the Rey–Osterrieth complex figure (copy and memory), and free and cued selective reminding test**. *Archives of Clinical Neuropsychology* 2009, **24**(4):371-393.

5. Dion M, Potvin O, Belleville S, Ferland G, Renaud M, Bherer L, Joubert S, Vallet GT, Simard M, Rouleau I: **Normative data for the Rappel libre/Rappel indicé à 16 items (16-item Free and Cued Recall) in the elderly Quebec-French population**. *The Clinical neuropsychologist* 2015, **28**(sup1):1-19.

6. Welsh KA, Butters N, Mohs RC, Beekly D, Edland S, Fillenbaum G, Heyman A: **The Consortium to Establish a Registry for Alzheimer's Disease (CERAD). Part V. A normative study of the neuropsychological battery**. *Neurology* 1994, **44**(4):609-614.

7. Aebi C: **Validierung der neuropsychologischen Testbatterie CERAD-NP: eine Multi-Center Studie**. University_of_Basel; 2002.

8. Wechsler D: **Weschler Memory Scale—3rd Edition**. In*.*: The Psychological Corporation New York, NY; 1997.

9. Pearson: **Repeatable Battery for the Assessment of Neuropsychological Status (RBANS)™**. In*.*; 2008.

10. Van der Elst W, Van Boxtel MP, Van Breukelen GJ, Jolles J: **Normative data for the Animal, Profession and Letter M Naming verbal fluency tests for Dutch speaking participants and the effects of age, education, and sex**. *Journal of the International Neuropsychological Society : JINS* 2006, **12**(1):80-89.

11. Novelli G, Papagno C, Capitani E, Laiacona M: **Tre test clinici di ricerca e produzione lessicale. Taratura su sogetti normali**. *Archivio di psicologia, neurologia e psichiatria* 1986.

12. Marien P, Mampaey E, Vervaet A, Saerens J, De Deyn PP: **Normative data for the Boston Naming Test in native Dutch-speaking Belgian elderly**. *Brain and language* 1998, **65**(3):447-467.

13. Peña-Casanova J, Quiñones-Úbeda S, Gramunt-Fombuena N, Aguilar M, Casas L, Molinuevo JL, Robles A, Rodríguez D, Barquero MS, Antúnez C: **Spanish Multicenter Normative Studies (NEURONORMA Project): norms for Boston naming test and token test**. *Archives of Clinical Neuropsychology* 2009, **24**(4):343-354.

14. Schmand B, Houx, P., de Koning, I., : **Norms for Stroop Color Word test, Trail making test, and Story recall of Rivermead Behavioural Memory Test**. In*.* De sectie Neuropsychologie van het Nederlands Instituut van Psychologen; 2003.

15. Van der Elst W, Van Boxtel MP, Van Breukelen GJ, Jolles J: **The Stroop color-word test: influence of age, sex, and education; and normative data for a large sample across the adult age range**. *Assessment* 2006, **13**(1):62-79.

16. Caffarra P, Vezzadini G, Dieci F, Zonato F, Venneri A: **Rey-Osterrieth complex figure: normative values in an Italian population sample**. *Neurological Sciences* 2002, **22**(6):443-447.
