# Supplementary material for: The EMIF-AD Multimodal Biomarker Discovery study: design, methods and cohort characteristics
Source: Alzheimers Res Ther. 2018 Jul 6;10:64. doi: 10.1186/s13195-018-0396-5 (PMC6035398; doi:10.1186/s13195-018-0396-5)
Supplement: Supplementary file 3 — Table S3. Biomarker protocol information for each cohort. Protocol information for PET, CSF and plasma biomarker collection (DOCX 100 kb) [file 13195_2018_396_MOESM3_ESM.docx]

| **Biomarker protocol information for each cohort** | | | | | | | | | | | | |  |
| --- | --- | --- | --- | --- | --- | --- | --- | --- | --- | --- | --- | --- | --- |
|  | PET |  | CSF | | | | |  | Plasma | | | | |
| Cohort | SUV  cut-off |  | Local assay | Local Aβ_42_  cut-off | Freeze/thaw  n 0/1/2/3/4 | Freezing temp. | Average storage time |  | Freeze/thaw  n 0/1/2 | Freezing temp. | Fasting | Average  storage time | |
| Amsterdam (n=172) | - |  | INNOTEST ELISA | <640 | 168/2/0/0/0 | -80°C | 6.6 ± 1.6 |  | 170/0/0 | -80°C | No | 6.6 ± 1.6 | |
| Antwerp (n=149) | - |  | INNOTEST ELISA | <638.5 | 0/113/30/5/1 | -75°C | 7.0 ± 3.3 |  | 0/140/0 | -75°C | Variable | 7.0 ± 3.3 | |
| DESCRIPA (n=29) | - |  | INNOTEST ELISA | <550 | 29/0/0/0/0 | -80°C | 12.9 ± 0.9 |  | 29/0/0 | -80°C | NA | 12.9 ± 0.9 | |
| EDAR (n=204) | - |  | xMAP AlzBio3 | <389 | 192/0/0/0/0 | -80°C | 8.3 ± 0.6 |  | 199/0/0 | -80°C | Variable | 8.4 ± 0.8 | |
| GAP (n=40) | - |  | INNOTEST ELISA | <550 | 40/0/0/0/0 | -80°C | 5.4 ± 0.5 |  | 40/0/0 | -80°C | Yes | 5.4 ± 0.5 | |
| Gothenburg (n=95) | - |  | INNOTEST ELISA | <550 | Not contributed | Not contributed | Not contributed |  | 92/0/0 | -80°C | NA | 10.5 ± 3.4 | |
| IDIBAPS (n=120) | - |  | INNOTEST ELISA | <550/<500* | Not contributed | Not contributed | Not contributed |  | 73/47/0 | -80°C | NA | 6.8 ± 1.6 | |
| Lausanne (n=40) | - |  | INNOTEST ELISA | <690 | 40/0/0/0/0 | -80°C | 4.3 ± 0.9 |  | 40/0/0 | -80°C | Yes | 4.3 ± 0.9 | |
| Leuven (n=180) | >1.38 |  | Not collected |  | Not contributed | Not contributed | Not contributed |  | 163/0/0 | -80°C | No | 5.0 ± 1.9 | |
| Pharmacog (n=147) | - |  | INNOTEST ELISA | <550 | 0/144/0 | -80°C | 4.2 ± 0.3 |  | 0/144/0 | -80°C | Yes | 4.3 ± 0.5 | |
| Sant Pau (n=45) | - |  | INNOTEST ELISA | <550 | Not contributed | Not contributed | - Not contributed |  | 45/0/0 | -80°C | Variable | 3.6 ± 1.1 | |
| *Two cut-off values available since samples were analyzed in two batches. Abbreviations: Aβ = amyloid-beta, CSF = cerebrospinal fluid, NA = not applicable, PET = positron emission tomography, temp. = temperature, SUVR = standardized uptake value. | | | | | | | | | | | | |  |
