# Supplementary material for: The EMIF-AD Multimodal Biomarker Discovery study: design, methods and cohort characteristics
Source: Alzheimers Res Ther. 2018 Jul 6;10:64. doi: 10.1186/s13195-018-0396-5 (PMC6035398; doi:10.1186/s13195-018-0396-5)
Supplement: Supplementary file 4 — Table S4. Ethical approval committee of each center. Ethical approval committees in each of the participating centers (DOCX 109 kb) [file 13195_2018_396_MOESM4_ESM.docx]

| **Ethical approval committee of each center** | | | |
| --- | --- | --- | --- |
| **Center** | **Part of multicenter** | **Country** | **Approval Committee** |
| Aristotle University, Thessaloniki | DESCRIPA, EDAR, Pharmacog | Greece | Aristotle University of Thessaloniki Medical School Ethics Committee |
| Central Institute for Mental Health, Mannheim | EDAR | Germany | Ethics Committee of the Medical Faculty Mannheim, University of Heidelberg |
| GAP, San Sebastian | - | Spain | Ethic and Clinical Research Committee Donostia |
| Hôpital Timone Adultes, Marseille | Pharmacog | France | Ethics committee Inserm and Aix Marseille University |
| Hospital Clínic de Barcelona IDIBAPS | Pharmacog | Spain | The Healthcare Ethics Committee of the Hospital Clínic |
| Hospital de la Santa Creu i Sant Pau, Barcelona | EDAR | Spain | Central Clinical Research and Clinical Trials Unit (UICEC Sant Pau) |
| INSERM, Toulouse | Pharmacog | France | INSERM Ethical Committee |
| IRCCS-FBF, Brescia | Pharmacog | Italy | Ethic Committee of the IRCCS San Giovanni di Dio FBF |
| IRCCS-SDN, Napels | Pharmacog | Italy | Comitato Etico IRCCS Pascale - Napoli |
| Karolinska Institutet, Stockholm | EDAR | Sweden | Ethics Committee at Karolinska Institutet |
| Katholieke Universiteit, Leuven | EDAR | Belgium | Ethische commissie onderzoek UZ/KU Leuven |
| Lausanne University Hospital, Lausanne | - | Switzerland | Research Ethics Committee Lausanne University Hospital |
| Maastricht University, Maastricht | DESCRIPA, EDAR | Netherlands | Medical ethical committee Maastricht University Medical Center |
| Rigshospitalet, Copenhagen | EDAR | Denmark | Committee on Health Research Ethics, Region of Denmark |
| University of Mediterranean, Marseille | Pharmacog | France | Ethics committee of Mediterranean University |
| University of Lille, Lille | Pharmacog | France | University of Lille Ethics committee |
| University of Leipzig, Leipzig | Pharmacog | Germany | Ethical Committee at the Medical Faculty, Leipzig University |
| University of Essen, Essen | Pharmacog | Germany | Ethical Committee at the Medical Faculty, University Hospital Essen |
| University of Antwerp, Antwerp | - | Belgium | Ethics committee University of Antwerp |
| University of Genoa, Genoa | Pharmacog | Italy | Ethical Committee of University of Genoa |
| University of Gothenburg, Gothenburg | - | Sweden | Ethics Committee, University of Gothenburg |
| University of Perugia, Perugia | Pharmacog | Italy | Human ethics Committee of the University of Perugia |
| VU Medical Center, Amsterdam | EDAR, Pharmacog | Netherlands | Medical ethics committee VU Medical Center |
